# Long-term Cognitive Trajectory After Total Joint Arthroplasty

**DOI:** 10.1001/jamanetworkopen.2022.41807

**Published:** 2022-11-14

**Authors:** Maria Vassilaki, Walter K. Kremers, Mary M. Machulda, David S. Knopman, Ronald C. Petersen, Mariana L. Laporta, Daniel J. Berry, David G. Lewallen, Hilal Maradit Kremers

**Affiliations:** 1 Department of Quantitative Health Sciences, Mayo Clinic, Rochester, Minnesota; 2 Department of Psychiatry and Psychology, Mayo Clinic, Rochester, Minnesota; 3 Department of Neurology, Mayo Clinic, Rochester, Minnesota; 4 Department of Anesthesiology and Perioperative Medicine, Mayo Clinic, Rochester, Minnesota; 5 Department of Orthopedic Surgery, Mayo Clinic, Rochester, Minnesota

## Abstract

**Importance:**

Individuals with total joint arthroplasty (TJA) have long-term exposure to metal-containing implants; however, whether long-term exposure to artificial implants is associated with cognitive function is unknown.

**Objective:**

To compare long-term cognitive trajectories in individuals with and without TJA.

**Design, Setting, and Participants:**

This population-based cohort study assessed serial cognitive evaluations of 5550 participants (≥50 years of age) from the Mayo Clinic Study of Aging between November 1, 2004, and December 31, 2020.

**Exposures:**

Total joint arthroplasty of the hip or the knee.

**Main Outcomes and Measures:**

Linear mixed-effects models were used to compare the annualized rate of change in global and domain-specific cognitive scores in participants with and without TJA, adjusting for age, sex, educational level, apolipoprotein E ε4 carrier status, and cognitive test practice effects.

**Results:**

A total of 5550 participants (mean [SD] age at baseline, 73.04 [10.02] years; 2830 [51.0%] male) were evaluated. A total of 952 participants had undergone at least 1 TJA of the hip (THA, n = 430) or the knee (TKA, n = 626) before or after entry into the cohort. Participants with TJA were older, more likely to be female, and had a higher body mass index than participants without TJA. No difference was observed in the rate of cognitive decline in participants with and without TJA until 80 years of age. A slightly faster cognitive decline at 80 years or older and more than 8 years from surgery was observed (b = −0.03; 95% CI, −0.04 to −0.02). In stratified analyses by surgery type, the faster decline was observed primarily among older participants with TKA (b = −0.04; 95% CI, −0.06 to −0.02).

**Conclusions and Relevance:**

In this cohort study, long-term cognitive trajectories in individuals with and without TJA were largely similar except for a slightly faster decline among the oldest patients with TKA; however, the magnitude of difference was small and of unknown clinical significance.

## Key Points

**Question:**

Is total joint arthroplasty associated with a faster cognitive decline later in life?

**Findings:**

In a cohort study of 5500 participants, no difference was found in the rate of cognitive decline in individuals with and without joint arthroplasty until 80 years of age. A slightly faster cognitive decline was observed at 80 years or older and more than 8 years after surgery, primarily among patients undergoing total knee arthroplasty.

**Meaning:**

Long-term cognitive trajectories appear to be largely similar in individuals with and without total joint arthroplasty, except for a small difference of unclear significance later in life.

## Teaser

This cohort study examines whether patients who have undergone total joint arthroplasty experience a faster cognitive decline than those without total joint arthroplasty.

## Introduction

Total joint arthroplasty (TJA) is the most common surgery in the US, with nearly 8 million Americans currently living with artificial hip and/or knee joints.^1^ Patients with TJA have long-term exposure to artificial metal-containing implants. Approximately half of patients with TJA undergo surgery before 65 years of age, and their life expectancy equates to long-term metal exposure of more than 20 years. Even though the duration of exposure is shorter for older patients with TJA, concomitant comorbidities could potentially make them more vulnerable to systemic effects. Thus far, we have little insight with respect to the safety and potential systemic effects of such long-term exposure.

Some long-term mortality studies^2,3^ and anecdotal case reports^4,5,6,7,8,9,10,11,12^ suggest an excess risk of neurotoxicity in patients undergoing total hip arthroplasty (THA) and total knee arthroplasty (TKA), with supportive findings from animal studies and occupational exposures.^13,14,15,16,17,18,19,20,21,22,23^ Symptoms reported in case reports include poor concentration, memory loss, and impaired attention or executive function in TJA patients.^4,5,6,7,8,9,10,11,12^ If neurotoxicity were associated with TJA, it could manifest many years after TJA. However, cognitive impairment is common in older adults, and minor alterations in cognition could be attributed to aging, obscuring the potential contribution of TJA. Therefore, establishing causality is particularly challenging with the long induction period of TJA exposure and because of the increased incidence of dementia in older adults. We examined the association of TJA with long-term changes in cognitive performance in a large population-based cohort with serial clinical and cognitive measures.

## Methods

Study protocols were approved by the Mayo Clinic and Olmsted Medical Center institutional review boards, and participants provided written informed consent before participation. In the case of participants with cognitive impairment sufficient to interfere with capacity, assent was obtained from a legally authorized representative. This study followed the Strengthening the Reporting of Observational Studies in Epidemiology (STROBE) reporting guideline.

### Study Population

We used data from the Mayo Clinic Study of Aging (MCSA).^24,25^ The MCSA is a prospective, population-based cohort study initiated in 2004 in Olmsted County, Minnesota, to investigate the epidemiology and risk factors for mild cognitive impairment. The recruitment and follow-up of MCSA participants are still ongoing, with serial cognitive evaluations every 15 months. The present study included data from a total of 22 630 study visits with cognitive testing from November 1, 2004, through December 31, 2020.

The MCSA evaluations are performed every 15 months using the same protocol. Each participant is evaluated by a study coordinator and a physician and undergoes neuropsychological testing by a psychometrist. During the coordinator's interview, the participant and an informant are questioned about memory and everyday function. The participant also completes the Beck Depression Inventory II^26^ and Beck Anxiety Inventory.^27^ The Short Test of Mental Status,^28^ a medical history review, and a neurologic examination are performed by a physician. Nine neuropsychological tests are administered to assess cognitive performance in 4 domains (ie, memory, attention/executive, language, and visuospatial abilities).^24,25^ Data collection also includes demographic information (including race and ethnicity as selected by the participant), medical history, educational level, and apolipoprotein E (*APOE*) ε4 genotyping using standard methods.^29^ The *APOE* ɛ4 carriers included participants with 1 or 2 copies of the ɛ4 allele (ie, ɛ2 ɛ4, ɛ3 ɛ4, ɛ4 ɛ4).

### TJA (Exposure) Information

The medical records of the MCSA participants with a history of THA and TKA were reviewed manually by trained nurse abstractors. Medical record review included clinical notes, operative reports, radiology reports, and patient-reported information. Data were abstracted on dates, types, indications for TJA surgery, and the specific type of hip and knee implants. Manually collected data were further supplemented by implant information from an institutional TJA registry. The implants (all components) were then classified according to composition of materials as chrome-cobalt, titanium, stainless steel, tantalum, ceramic, and polyethylene. A total of 73 participants with missing surgical information (ie, exact dates, locations, or surgery type not found) were excluded.

### Statistical Analysis

The raw scores for the neuropsychological tests in each cognitive domain were *z*-scored and averaged to create domain-specific cognitive *z* scores (ie, memory, attention/executive, language, and visuospatial skills); a global cognitive *z* score for overall cognitive performance was calculated by averaging the 4 domain-specific *z* scores. All patients had at least 1 domain-specific cognitive *z* score in all visits, but a global cognitive *z* score could not be calculated in 8% of the visits because at least 1 domain-specific score was missing.

Initial analyses considered TJA as the exposure of interest. Because of differences in implants, anatomy, movement, and potential friction, the main analyses were stratified by joint type. We modeled cognitive *z* scores using a mixed-effects model with fixed effects for a difference at time of surgery as well as rate of change in *z* score following separately for THA and TKA, as well as fixed effects for age, sex, educational level, *APOE* ε4 genotype, and practice effect. Rate of change after TJA was fit using a linear spline with knots at 2, 4, and 8 years after surgery. This spline allows for a difference between those with and without TJA immediately after TJA surgery through an intercept term plus a cumulative effect as a function of time measured in years since TJA using slope terms. Before any surgery, time since surgery was considered to be 0, and there was no model effect of surgery. The cumulative TJA exposure was recalculated at each visit with cognitive scores. We also fit linear splines for cognition as a function of age, separately for males and females, with knots at 5-year intervals (ie, 55 years, 60 years, and so on). For years of education, we used linear spline with knots at 12 and 16 years in which 8 years of education was imputed for years less than 8 and 20 years of education was imputed for years greater than 20. The association with the *APOE* ε4 effect was fit using a linear spline as a function of age with knots at 5-year intervals. For practice effect, we included specific terms for the first and second visit and a linear spline for years since the first study visit with knots at 4 and 8 years from the first visit. In addition, random patient-specific effects were included for intercept, rate of change from MCSA visit 1, and rate of change since THA and TKA, which were allowed to be correlated.

We also investigated the impact of implant metal types by fitting separate linear spline functions for implants made of only chrome-cobalt, only titanium, or chrome-cobalt and titanium together. Implants of other types (eg, tantalum, ceramic) and patients with metal-on-metal THA implants (n = 15) were too few for robust analyses. Metal–type-specific analyses were performed based on metal type of the first TJA surgery, and data were removed (censored) from the analysis data set on implantation of an implant of a different metal type. In sensitivity analyses, we analyzed data sets excluding visits with both a THA and TKA and data sets excluding visits more than 16 years after the first TJA. We further performed sensitivity analyses including terms for body mass index (BMI), gait speed, and the components of the Charlson Comorbidity Index (excluding dementia) (intercept and association with time) to the mixed model. We used the Fieller theorem to derive 95% CIs for ratios of rates of cognitive change.^30^ All analyses were considered statistically significant at a 2-sided *P* < .05. Analyses were performed using SAS software, version 9.4 (SAS Institute Inc). No adjustments were made for multiple comparisons.

## Results

### Study Population

The study cohort included 5550 participants. Mean (SD) age at baseline was 73.04 (10.02) years, 2830 participants (51.0%) were men and 2720 (49.0%) were women, and 5406 (97.4%) were White. The mean (SD) follow-up of the entire cohort was 4.17 (3.64) years, and the median (range) was 3.88 (0.00-14.51) years. A total of 724 participants had a history of at least 1 surgery before their first MCSA visit (baseline), and 228 participants had their first surgery during follow-up but before the last MCSA cognitive evaluation (eTable 1 in the Supplement). Of the 952 participants with TJA, 430 had at least 1 THA and 626 had at least 1 TKA. At baseline, 724 participants with a history of TJA were significantly older, more likely to be female, and had a higher BMI, higher Beck Depression Inventory II and Beck Anxiety Inventory scores, slower gait speed, and higher Charlson Comorbidity Index than the participants without TJA at the MCSA baseline (Table 1). The magnitude of differences adjusted for age were small relative to the SD of the respective characteristic. Adjusted for age, the baseline global and domain-specific cognitive *z* scores had no significant differences (Table 1).

**Table 1.** Baseline Characteristics of the 5550 Study Participants by TJA Status

Characteristic	No. of participants with data	Participants with TJA at baseline (n = 724)^a^	Participants without TJA at baseline (n = 4826)^a^	Difference (SE) adjusted for age^b^	*P* value
Age, mean (SD), y	5550	76.96 (7.81)	72.45 (10.18)	4.52 (0.39)	<.001
Sex					
Male	5550	326 (45.03)	2504 (51.89)	−0.08 (0.02)	<.001
Female		398 (55.00)	2322 (48.11)		
Length of education, mean (SD), y	5550	14.01 (2.78)	14.36 (2.78)	−0.10 (0.11)	.36
*APOE* ε24/ε34/ε4	5550	203 (28.0)	1319 (27.3)	0.01 (0.02)	.46
BMI, mean (SD)	5437	30.69 (6.13)	28.21 (5.32)	2.95 (0.22)	<.001
BDI II total score, mean (SD)	5446	5.49 (5.22)	4.88 (5.03)	0.42 (0.20)	.04
BAI total score (range, 0-63), mean (SD)	5536	3.31 (4.57)	2.86 (4.32)	0.43 (0.18)	.014
Gait speed, mean (SD), m/s to walk 25 ft	5125	0.99 (0.27)	1.12 (0.26)	−0.08 (0.01)	<.001
Charlson Comorbidity Index, mean (SD)	5549	3.89 (3.18)	2.99 (3.04)	0.41 (0.12)	<.001
Cognitive measures/status at baseline, mean (SD)					
Global *z* score^c^	5200	0.24 (1.07)	0.54 (1.12)	0.01 (0.04)	.83
Memory *z* score	5522	0.17 (1.10)	0.41 (1.13)	0.01 (0.04)	.73
Attention/executive *z* score	5359	0.27 (1.08)	0.53 (1.07)	0.02 (0.04)	.58
Visuospatial *z* score	5341	0.12 (1.02)	0.38 (1.07)	−0.03 (0.04)	.45
Language *z* score	5401	0.17 (1.02)	0.35 (1.07)	0.04 (0.04)	.33

Abbreviations: *APOE*, apolipoprotein E; BAI, Beck Anxiety Inventory; BDI II, Beck Depression Inventory II; BMI, body mass index (calculated as weight in kilograms divided by height in meters squared); TJA, total joint arthroplasty.

^a^ Data are presented as number (percentage) of patients unless otherwise indicated.

^b^ Differences between the TJA and non-TJA groups are given as mean differences for numerical variables and as odds ratios for categorical variables; all variables (except age) are adjusted for age.

^c^ Cognitive *z* scores computed after scaling raw cognitive test scores (mean [SD], 0 [1]) using data for cognitively unimpaired participants at baseline. Domain-specific *z* scores are summed and scaled to obtain the global *z* score.

### Change in Cognitive *z* Scores in Participants With THA and TKA

The mean annual rate of global cognitive decline among participants with TJA did not differ significantly from participants without TJA until 80 years of age (Table 2). However, participants with TJA who were 80 years or older and more than 8 years after surgery experienced a slightly faster cognitive decline thereafter (estimate, −0.03; 95% CI, −0.04 to −0.02; *P* < .001).

**Table 2.** Difference in Annual Rate of Change on Global Cognitive *z* Score According to Time Since Surgery

Time since surgery, y	b (95% CI)^a^		
	TJA	THA	TKA
**<80 y of age**			
0-2	−0.01 (−0.05 to 0.04)	0.03 (−0.05 to 0.10)	−0.03 (−0.09 to 0.03)
2-4	−0.004 (−0.04 to 0.03)	−0.03 (−0.08 to 0.02)	0.01 (−0.03 to 0.06)
4-8	0.002 (−0.02 to 0.02)	0.01 (−0.02 to 0.04)	−0.004 (−0.03 to 0.02)
>8	−0.01 (−0.02 to 0.006)	−0.007 (−0.03 to 0.02)	−0.01 (−0.04 to 0.01)
**≥80 y of age**			
0-2	0.02 (−0.04 to 0.07)	0.04 (−0.05 to 0.12)	0.002 (−0.07 to 0.08)
2-4	0.001 (−0.04 to 0.04)	0.01 (−0.05 to 0.07)	−0.005 (−0.06 to 0.05)
4-8	−0.005 (−0.02 to 0.02)	0.005 (−0.03 to 0.04)	−0.01 (−0.04 to 0.02)
>8	−0.03 (−0.04 to −0.02)	−0.01 (−0.04 to 0.01)	−0.04 (−0.06 to −0.02)

Abbreviations: THA, total hip arthroplasty; TJA, total joint arthroplasty; TKA, total knee arthroplasty.

The Figure illustrates global cognitive *z* score trajectories as a function of age and sex in participants with and without THA or TKA. Differences in the annual rate of change in global cognitive *z* scores according to years from THA and TKA are shown in Table 2. For participants who had THA or TKA at 60 years of age (Figure, A and B), global cognitive *z* scores over time did not differ significantly from participants without THA or TKA for up to 14.5 years of follow-up (up to 48.4 years since TJA). For participants who had THA at 70 or 80 years of age (Figure, C and E), the rate of cognitive decline did not differ significantly from participants without THA. The difference in annual rate of change at different years in participants with and without THA were small and ranged from −0.007 to 0.04 (Table 2). However, participants who had TKA at 70 years (Figure, D) experienced a slightly faster decline in global cognitive *z* scores after 8 years from their surgery and at 80 years or older with a rate −0.04 per year faster than the participants without TKA (Table 2). A similar pattern was observed for memory and attention/executive *z* scores (eTable 2 in the Supplement). For an 80-year-old patient who was more than 8 years out from TKA surgery, a −0.04 per-year difference in global *z* score corresponded to a 35.0% (95% CI, 17.6%-56.0%) faster decline or incurring 3.2 additional months of cognitive decline. Of note, at any point in time, the association for TJA is the result of the cumulative association for TJA from the time of surgery up to that point in time (cumulative exposure), calculated by summing the associations over time since surgery. There was faster rate of cognitive decline in the participants with TKA compared with non-TKA at earlier time points, but this finding was not significant. For attention/executive *z* scores, participants with TKA had slightly lower estimated mean scores than participants without TKA beginning at 14 years from their surgery and at 80 years or older (ie, with a rate −0.28 per year faster than the participants without TKA; *P* = .03) and increasing thereafter (ie, after 16 years from their surgery and at 80 years or older with a rate −0.37 per year faster than the participants without TKA; *P* = .01).

**Figure.**
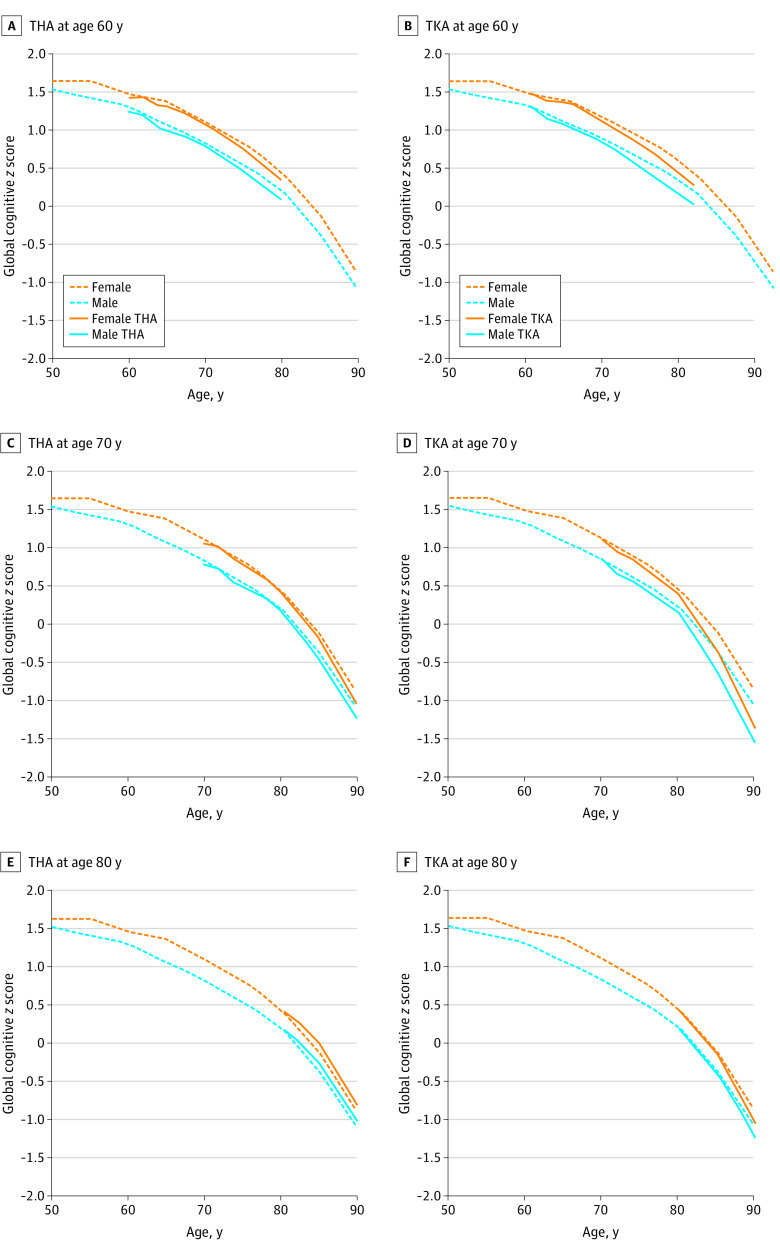
Global Cognitive *z* Score Trajectories From Linear Mixed Models by Age at Total Hip Arthroplasty (THA) and Total Knee Arthroplasty (TKA) The lines correspond to mean global *z* scores for persons with 12 years of education.

Findings persisted in sensitivity analyses after adjusting for BMI, gait speed, and the Charlson Comorbidity Index. In participants who were 80 years or older and more than 8 years after surgery, the rate of decline in the global *z* score was faster for TJA overall (−0.03; 95% CI, −0.04 to −0.01; *P* = .002), THA (−0.03; 95% CI, −0.05 to −0.001; *P* = .046), and TKA (−0.03; 95% CI, −0.04 to −0.009; *P* = .004). However, residual confounding is likely because these covariates were measured at the MCSA baseline and not at the time of TJA surgery, which was several years before the MCSA baseline in most cases.

### Metal Type of TJA Implants

Implant information was available for 1573 of the 1592 TJAs in 952 participants, with 1129 implants (71.8%) containing chrome-cobalt, 1029 (65.4%) titanium, 30 (1.9%) stainless steel, 136 (8.6%) oxinium, and 16 (0.6%) tantalum (eTable 1 in the Supplement). Analyses fitted separate associations for chrome cobalt (n = 219), titanium (n = 172), and combined chrome cobalt and titanium (n = 390) at first surgery and found slightly accelerated decreases in global (Table 3), memory, and attention/executive *z* scores for participants with titanium or combined chrome cobalt and titanium TKA implants who were 80 years or older and more than 8 years from surgery. This finding was most prominent for TKA implants that contained titanium (global: −0.07; 95% CI, −0.12 to −0.03; memory: −0.05; 95% CI, −0.10 to 0.002; attention/executive: −0.07; 95% CI, −0.12 to −0.02), intermediate for those with combined implants (global: −0.03; 95% CI, −0.06 to −0.006; memory: −0.03; 95% CI, −0.06 to 0.001; attention/executive: −0.04; 95% CI, −0.07 to −0.01), and nonsignificant for chrome cobalt implants (global: −0.02; 95% CI, −0.06 to 0.03; memory: −0.03; 95% CI, −0.09 to 0.02; attention/executive: −0.02; 95% CI, −0.07 to 0.03). The remaining associations for the combinations of metal type and cognitive domains were generally not notable except for the global score for patients with titanium TKA implants at more than 8 years after surgery and younger than 80 years (global: −0.07; 95% CI, −0.14 to −0.008) and with chrome-cobalt hip implants after 8 years and 80 years or older (global: −0.05; 95% CI, −0.10 to −0.001).

**Table 3.** Difference in Annual Rate of Change on Global Cognitive *z* Score According to Metal Composition of Implants and Time Since Surgery

Time since surgery, y	b (95% CI)^a^	
	THA	TKA
**Chrome-cobalt implants in those <80 y of age**		
0-2	−0.62 (−1.31 to 0.06)	−0.11 (−0.20 to −0.01)
2-4	−0.02 (−0.27 to 0.23)	0.06 (−0.01 to 0.13)
4-8	0.04 (−0.09 to 0.18)	−0.02 (−0.06 to 0.02)
>8	−0.06 (−0.13 to 0.02)	−0.05 (−0.12 to 0.02)
**Chrome-cobalt implants in those ≥80 y of age**		
0-2	0.06 (−0.34 to 0.46)	0.07 (−0.07 to 0.21)
2-4	0.17 (−0.02 to 0.36)	0.02 (−0.07 to 0.10)
4-8	0.006 (−0.07 to 0.08)	−0.002 (−0.05 to 0.04)
>8	−0.05 (−0.10 to −0.001)	−0.02 (−0.06 to 0.03)
**Titanium implants in those <80 y of age**		
0-2	0.04 (−0.10 to 0.18)	0.04 (−0.08 to 0.16)
2-4	0.002 (−0.12 to 0.12)	0.04 (−0.06 to 0.14)
4-8	−0.06 (−0.15 to 0.04)	0.02 (−0.05 to 0.09)
>8	−0.08 (−0.23 to 0.08)	−0.07 (−0.14 to −0.008)
**Titanium implants in those ≥80 y of age**		
0-2	0.12 (−0.09 to 0.32)	−0.05 (−0.21 to 0.12)
2-4	0.20 (−0.03 to 0.43)	−0.01 (−0.12 to 0.10)
4-8	0.13 (−0.06 to 0.33)	−0.03 (−0.08 to 0.03)
>8	0.06 (−0.08 to 0.20)	−0.07 (−0.12 to −0.03)
**Chrome-cobalt and titanium implants in those <80 y of age**		
0-2	0.04 (−0.06 to 0.13)	0.006 (−0.10 to 0.11)
2-4	−0.06 (−0.12 to 0.01)	−0.06 (−0.14 to 0.01)
4-8	0.006 (−0.04 to 0.05)	−0.001 (−0.04 to 0.04)
>8	0.02 (−0.03 to 0.07)	0.000 (−0.04 to 0.04)
**Chrome-cobalt and titanium implants in those ≥80 y of age**		
0-2	−0.01 (−0.12 to 0.09)	0.002 (−0.13 to 0.13)
2-4	0.02 (−0.05 to 0.10)	−0.04 (−0.13 to 0.05)
4-8	−0.008 (−0.05 to 0.04)	−0.01 (−0.05 to 0.04)
>8	0.003 (−0.04 to 0.04)	−0.03 (−0.06 to −0.006)

Abbreviations: THA, total hip arthroplasty; TKA, total knee arthroplasty.

^a^ Adjusted for age, sex, educational level, test naivety (whether the participant had previously taken the cognitive tests), and apolipoprotein ε4 status.

## Discussion

In this cohort study, we examined the association between TJA surgery and long-term cognitive trajectories in a population-based cohort with serial comprehensive cognitive measurements. No significant association was found between TJA surgery and the rate of cognitive decline, except for patients with TKA who were 80 years or older with more than 8 years of exposure. However, the difference was small, and the clinical significance is unclear. The observed difference corresponded to 3.2 additional months of cognitive decline in patients with TKA. Although there is no universally accepted level that would be considered a clinically meaningful delay in decline, some authors^31^ asserted that it should be a 1-year delay, and others have found that a delay of less than 3 months would not be clinically evident.^32^

Our study provides observational evidence beyond findings of previous case reports.^4,5,6,7,8,9,10,11,12^ Systemic distribution of wear debris from metal implants is unavoidable. Friction and corrosion over time generate wear debris that may result in increased local and systemic concentrations of metal nanoparticles and soluble metal ions.^16,17,18^ Poor concentration, impaired attention or executive function, and memory loss were symptoms reported in patients with TJA in previous case reports.^4,5,6,7,8,9,10,11,12^ Other findings include peripheral neuropathy, hearing loss, visual impairment, and neuropsychiatric symptoms.^8,12,33^ However, studies with long-term follow-up are limited, and it is unknown how patients react to these metal ions, what or how severe these reactions would be, and whether there are systemic effects.^19,20,21,22,23,34^ Titanium dioxide, a frequently used biomaterial (eg, in orthopedic implants), is considered an inert and benign compound, and its ability to reach the nervous system is for the most part unknown in humans.^23^ In animal studies, rats with titanium implants or intravascularly treated with titanium dioxide nanoparticles had memory impairments, and intravascularly treated rats had titanium in their brain.^23^ In addition, treating a human blood-brain barrier (BBB) in vitro model to sera of rats with titanium implants increased the BBB permeability, even though titanium was not detected in the brain of the rats, suggesting the hypothesis that circulating factors (eg, inflammation factors) might have an effect on the integrity of the BBB making the penetration of toxins in the central nervous system easier.^23^ However, research is still limited and further studies are warranted.

Even though exposure duration might be relatively shorter for older patients with TJA, concomitant comorbidities could make them more vulnerable to systemic effects. It is plausible that adverse reactions to metal debris result from the intrinsic host-specific factors (ie, host response to metal debris) and the metal debris (ie, extrinsic factor).^35^ Additional mechanistic studies with systematic measurement of metals in the blood, histopathologic studies from TJA revision sites and other organs, and neuroimaging and neuropathologic studies would help to better interpret the trajectories observed in this observational study.

The long-term cognitive effects of general anesthesia and surgery in older adults is still under discussion.^36,37^ Previous analyses of this cohort suggested that older adults with surgery in the 20 years before the MCSA baseline (vs no exposure) had a subtle but greater decrease in global cognitive *z* scores.^36^ A more recent report^37^ suggested that major surgery is associated with only a small decline in cognitive performance (ie, <5 months of mean cognitive decline), which was less than the impact of major medical events or stroke. In the current study, we observed slightly faster cognitive decline in older participants with TKA and those with titanium implants, but the magnitude was very small and is unlikely to be detected clinically.

### Strengths and Limitations

This study has several strengths. Both TJA and cognitive impairment are common in older adults, and minor alterations in cognition could be attributed to normal aging, obscuring the potential contribution of TJA. The MCSA, a prospective, population-based study of cognitive aging with available serial comprehensive cognitive evaluation, provides a unique opportunity to study longitudinal trajectories over many years of follow-up. In addition, cognitive evaluations were blind to any prior history of TJA. The availability of an institutional TJA registry and access to original operative notes further allowed us to ascertain accurate information on surgical history and implant type and metallurgy.

Findings should nevertheless be considered in light of potential limitations. The comparison group, MCSA participants without TJA, do not necessarily represent how patients with TJA might have fared without the procedure, especially because higher levels of physical activity are associated with less pronounced cognitive decline.^38^ Blood metal measurements were not available because they were rarely measured clinically. We did not have information on TJA failure modes. Patients who underwent subsequent TJA revisions may have much higher metal levels because of wear of the implant surfaces or corrosion of modular connections. Because TJA is an elective procedure, it is offered to healthier patients (selection bias), whereas older individuals often have concomitant health conditions that could influence postsurgery recovery or be risk factors for late-life cognitive decline. Furthermore, patients with TJA at an older age may have had other arthritic joints that limited their mobility with consequent effects on cognitive status. Otherwise, in this study, no significant difference in mortality was found in the TJA and the non-TJA cohorts. We acknowledge that secular trends in cognitive decline have been observed, with later-born cohorts having higher baseline cognitive abilities and a less steep decline.^39^ Secular trends have not been studied in the MCSA cohort, but it is unlikely that such trends would account for the small differences we observed in only the participants with TKA. The observed cognitive differences could also be attributable to other underlying comorbidities, including osteoarthritis itself.^40,41^ It is possible that patients with multiple comorbidities or limited independence would be less willing to participate or would be more likely to drop out of the study during follow-up, underestimating the TJA–cognitive decline association if those who never enrolled had a steeper cognitive decline trajectory than those participating in the study and limiting generalizability. Finally, the MCSA is an observational study, so confounding by unmeasured factors cannot be excluded.

## Conclusions

In this cohort study, the long-term cognitive trajectories in individuals with and without TJA were largely similar except for a slightly faster decline among the oldest TKA patients. The magnitude of the difference was small and of unknown clinical significance.

## Notes

Supplement. **eTable 1.** TJA History and Implant Composition for 952 Participants With at Least One Surgery **eTable 2.** Difference in Annual Rate of Change on Domain-Specific *z* Scores According to Time Since Surgery
