# Supplementary material for: Long-term Cognitive Trajectory After Total Joint Arthroplasty
Source: JAMA Netw Open. 2022 Nov 14;5(11):e2241807. doi: 10.1001/jamanetworkopen.2022.41807 (PMC9664257; doi:10.1001/jamanetworkopen.2022.41807)
Supplement: Supplement. — eTable 1. TJA History and Implant Composition for 952 Participants With at Least One Surgery eTable 2. Difference in Annual Rate of Change on Domain-Specific z Scores According to Time Since Surgery [file jamanetwopen-e2241807-s001.pdf]

Supplementary Online Content

Vassilaki M, Kremers WK, Machulda MM, et al. Long-term cognitive trajectory after total joint arthroplasty. *JAMA Netw Open*. 2022;5(11):e2241807.
doi:10.1001/jamanetworkopen.2022.41807

eTable 1. TJA History and Implant Composition for 952 Participants With at Least One Surgery

eTable 2. Difference in Annual Rate of Change on Domain-Specific *z* Scores According to Time Since Surgery

This supplementary material has been provided by the authors to give readers additional information about their work.

© 2022 Vassilaki M et al. *JAMA Network Open.*

eTable 1. TJA History and Implant Composition for 952 Participants With at Least One Surgery

Patient characteristics	Participants with TJA at baseline N=724	Participants with first TJA during follow-up N=228	Total Participants with TJA N=952
Total hip arthroplasty (THA)	318 (44%)	98 (43%)	430 (45%)
Total knee arthroplasty (TKA)	468 (65%)	140 (61%)	626 (66%)
Metal composition (ever)			
Cobalt-chromium	547 (76%)	152 (67%)	699 (74%)
Titanium	478 (66%)	160 (70%)	638 (67%)
Stainless steel	10 (1%)	4 (2%)	14 (1.5%)
Oxinium/ceramic	39 (5%)	31 (14%)	70 (7%)
Tantalum	5 (0.7%)	0 (0%)	5 (0.5%)
**Surgery characteristics**	**TJA surgeries before baseline N=1139**	**TJA surgeries during follow-up N=304**	**Total TJA surgeries* N=1592**
Total hip arthroplasty (THA)	451 (40%)	119 (39%)	641 (40%)
Total knee arthroplasty (TKA)	688 (60%)	185 (61%)	951 (60%)
Metal composition			
Cobalt-chromium	928 (73%)	201 (66%)	1129 (72%)
Titanium	820 (65%)	209 (69%)	1029 (65%)
Stainless steel	21 (2%)	4 (2%)	30 (2%)
Oxinium/ceramic	94 (7%)	42 (14%)	136 (9%)
Tantalum	15 (1%)	1 (0.3%)	16 (1%)

* Implant metal composition was not available for 20 surgeries. 724 participants with a history of 1139 TJA surgery at baseline had 149 additional TJA surgeries during follow-up.

© 2022 Vassilaki M et al. *JAMA Network Open.*

eTable 2. Difference in Annual Rate of Change on Domain-Specific Z Scores According to Time Since Surgery

Years since surgery	THA b (95% CI)[a]	TKA b (95% CI)[a]
**z score Memory**		
<80 years of age		
0-2 y	0.041 (-0.061 to 0.143)	-0.035 (-0.118 to 0.048)
2-4 y	-0.006 (-0.083 to 0.071)	0.021 (-0.039 to 0.081)
4-8 y	-0.008 (-0.049 to 0.033)	0.002 (-0.030 to 0.034)
>8 y	0.008(-0.016 to 0.031)	0.003 (-0.024 to 0.029)
≥80 years of age		
0-2 y	0.014 (-0.103 to 0.131)	0.050 (-0.048 to 0.148)
2-4 y	-0.009 (-0.090 to 0.072)	-0.030 (-0.098 to 0.038)
4-8 y	0.010 (-0.032 to 0.052)	0.003 (-0.030 to 0.036)
>8 y	-0.015 (-0.042 to 0.012)	-0.035 (-0.056 to -0.013)
**z score Attention/Executive**		
<80 years of age		
0-2 y	0.061 (-0.032 to 0.153)	-0.024 (-0.099 to 0.051)
2-4 y	-0.057 (-0.126 to 0.011)	0.000 (-0.054 to 0.054)
4-8 y	0.018 (-0.018 to 0.054)	-0.007 (-0.036 to 0.022)
>8 y	-0.016 (-0.036 to 0.004)	-0.004 (-0.029 to 0.021)
≥80 years of age		
0-2 y	0.060 (-0.046 to 0.167)	0.005 (-0.0879 to 0.098)
2-4 y	-0.057 (-0.131 to 0.018)	0.026 (-0.0381 to 0.090)
4-8 y	-0.008 (-0.046 to 0.031)	-0.032 (-0.064 to -0.000)
>8 y	-0.003 (-0.028 to 0.022)	-0.047 (-0.068 to -0.026)
**z score Language**		
<80 years of age		
0-2 y	-0.020 (-0.115 to 0.075)	-0.051 (-0.129 to 0.026)
2-4 y	-0.023 (-0.095 to 0.049)	0.016 (-0.040 to 0.071)
4-8 y	0.006 (-0.031 to 0.043)	0.002 (-0.029 to 0.032)
>8 y	0.004 (-0.016 to 0.023)	-0.025 (-0.050 to -0.000)
≥80 years of age		
0-2 y	0.029 (-0.081 to 0.140)	0.030 (-0.064 to 0.124)
2-4 y	0.039 (-0.038 to 0.116)	-0.003 (-0.068 to 0.062)

Years since surgery	THA<br>b (95% CI)[a]	TKA<br>b (95% CI)[a]
4-8 y	0.018 (-0.022 to 0.057)	-0.022 (-0.054 to 0.009)
>8 y	-0.001 (-0.026 to 0.023)	-0.013 (-0.034 to 0.007)
**z score Visuospatial**		
<80 years of age		
0-2 y	0.044 (-0.058 to 0.146)	-0.014 (-0.097 to 0.069)
2-4 y	-0.022 (-0.097 to 0.053)	0.008 (-0.051 to 0.066)
4-8 y	0.025 (-0.012 to 0.063)	0.007 (-0.024 to 0.037)
>8 y	-0.024 (-0.042 to -0.005)	-0.018 (-0.039 to 0.004)
≥80 years of age		
0-2 y	0.028 (-0.088 to 0.144)	-0.038 (-0.140 to 0.063)
2-4 y	-0.009 (-0.088 to 0.070)	-0.004 (-0.072 to 0.065)
4-8 y	-0.013 (-0.053 to 0.027)	0.004 (-0.027 to 0.036)
>8 y	0.005 (-0.019 to 0.028)	-0.003 (-0.022 to 0.016)

[a] Adjusted for age, sex, education, test naïve (whether or not the participant had previously taken the cognitive tests), APOE ε4 status.
All estimates for THA and TKA are derived simultaneously from the same model.

© 2022 Vassilaki M et al. *JAMA Network Open.*
